# Single and Bunch Soliton Generation in Optical Fiber Lasers Using Bismuth Selenide Topological Insulator Saturable Absorber

**DOI:** 10.3390/nano13091538

**Published:** 2023-05-03

**Authors:** Hazlihan Haris, Tan Sin Jin, Malathy Batumalay, Ahmad Razif Muhammad, Jahariah Sampe, Arni Munira Markom, Huda Adnan Zain, Sulaiman Wadi Harun, Megat Muhammad Ikhsan Megat Hasnan, Ismail Saad

**Affiliations:** 1 Faculty of Engineering, Universiti Malaysia Sabah (UMS), Kota Kinabalu 88400, Sabah, Malaysia; megatikhsan@ums.edu.my; 2 School of Engineering, KDU University College, UOW Malaysia, Shah Alam 40150, Selangor, Malaysia; sj.tan@uow.edu.my; 3 KDU University College, UOW Malaysia, George Town 10400, Pulau Pinang, Malaysia; 4 Faculty of Data Science & IT, INTI International University, Nilai 71800, Negeri Sembilan, Malaysia; malathy.batumalay@newinti.edu.my; 5 Institute of Microengineering and Nanoelectronics (IMEN), Universiti Kebangsaan Malaysia (UKM), Bangi 43600, Selangor, Malaysia; a.razif@ukm.edu.my (A.R.M.); jahariah@ukm.edu.my (J.S.); 6 School of Electrical Engineering, College of Engineering, Universiti Teknologi MARA, Shah Alam 40450, Selangor, Malaysia; arnimunira@uitm.edu.my; 7 Department of Electrical Engineering, Faculty of Engineering, University of Malaya, Kuala Lumpur 50603, Malaysia; huda.adnan.727@gmail.com (H.A.Z.); swharun@um.edu.my (S.W.H.)

**Keywords:** soliton generation, fiber lasers, bismuth selenide, topological insulator, saturable absorber

## Abstract

In this work, we present the generation of two distinct types of soliton pulses using a Bismuth Selenide (Bi_2_Se_3_) saturable absorber (SA) synthesized in our laboratory. The soliton pulses were generated in two different laser cavity configurations, resulting in two types of solitons: a soliton pulse with Kelly sidebands and a bunched soliton pulse with peak-dip sidebands. Both solitons operated at the fundamental repetition rate—23.3 MHz (for the soliton with Kelly sidebands) and 13 MHz (for the bunched soliton with peak-dip sidebands). We observed that the accumulation of nonlinear phase shift from the added single mode fiber (SMF) split the single soliton pulse into 44 pulses in a bunched oscillation envelope. At the same time, peak-dip sidebands were imposed on the bunched soliton spectrum due to constructive and destructive interferences between soliton pulse and dispersive waves. The measured pulse width for both solitons were 0.63 ps (for the soliton with Kelly sidebands) and 1.52 ps (for the bunched soliton with peak-dip sidebands), respectively. Our results demonstrate the potential of Bi_2_Se_3_ SAs in generating different types of soliton pulses, which could have potential applications in various areas of optical communication and spectroscopy.

## 1. Introduction

The pulse laser was first observed in 1985 in solid-state lasers using dye lasers [1]. Since then, the technology has advanced rapidly, especially with the introduction of the Semiconductor Saturable Absorber Mirror (SESAM) in 1996 by U. Keller et al. [2]. Alternative pulsing methods were also demonstrated in Erbium Doped Fiber Lasers (EDFL) in 1992 via the Nonlinear Polarization Rotation (NPR) technique [3]. These advances have attracted tremendous interest among researchers due to their broad range of applications in optical communication, material processing, and medical fields [4,5,6].

Today, there are many types of pulses produced to cater to different applications, making it important to understand pulse dynamics and characteristics. Fiber lasers provide an excellent platform for the in-depth study of pulse dynamics. Various mode-locked pulses have been reported, including the generation of single soliton and multiple solitons. Initial works revealing single and multiple solitons were centered around artificial SA [7], namely nonlinear polarization rotation (NPR) [8,9,10,11], nonlinear amplifying loop mirror (NALM) [12], and spectral effect [13].

The understanding of single solitons in a fiber laser is relatively straightforward, but for multiple solitons, there are several mechanisms that cause multiple soliton formation, including wave-breaking, soliton peak clamping, spectral filtering, and soliton shaping of dispersive waves. Various pulse dynamics on multiple solitons dynamics were reported such as bunched soliton [14], bound soliton [15], and soliton rain [16], with careful consideration being given to light polarization, pump power, and the cavity design of a fiber laser. Additionally, various researchers have studied and described variations in soliton sidebands. While the soliton pulse is always accompanied by Kelly sidebands, other less common sidebands have also been reported, namely peak-dip and dip soliton sidebands. Han and Yun [17] experimentally observed three types of sidebands for the first time by adjusting the polarization state of light in the laser cavity via the NPR technique. They claimed that the formation of peak-dip and dip sidebands was attributed to periodic power variation. Du et al. [18] indicated that the dip-type soliton was due to destructive interference between dispersive waves and solitons in their theoretical and numerical work.

Research is still ongoing with these two pulsing techniques, but lately, the research on multiple pulse dynamics and the variation of soliton sidebands has intensified with the discovery of novel materials to replace SESAM. Single soliton pulses, as well as various multiple soliton pulses and their profiles, were demonstrated in fiber lasers with the utilization of saturable absorbers (SA), including graphene [19,20,21], graphene oxide [22,23], carbon nanotube [24,25,26,27,28], transition metal dichalcogenide (TMD) [29,30,31], Topological Insulator (TI) [32,33,34], Black Phosphorus (BP) [35,36,37], and MXene [38,39,40]. The focus of researches has shifted to real SA from artificial SA due to their broadband saturable absorption properties, fast relaxation time, low saturation fluency, and simple fabrication process. Disordered multiple soliton, bunched soliton, and higher order harmonic mode locking were obtained by fine-tuning the light polarization in a work conducted by Meng et al. [20] using graphene SA. Liang et al. [41] confirmed the generation and transformation of bound soliton to bunched soliton when the pump power was increased through the utilization of MoS2 SA. Bound soliton evolved into bunched soliton to neutralize the accumulation of nonlinear effects in the laser cavity with the increment of pump power. Additionally, Yu et al. [42] coated BP on a tapered fiber to achieve single soliton pulse and bunched soliton with a total of 25 solitons in a single bunch at 37th harmonics. The single fundamental soliton grew into soliton bunches as the pump power was increased up to 322 mW. On the other hand, Guo et al. [43] experimentally studied on six different types of unusual sidebands by adjusting the pump strength and light polarization with the aid of WS2 SA.

In this work, we report the formation of single soliton pulses and bunch solitons utilizing Bi_2_Se_3_ (Bismuth Selenide) as an SA in two different laser cavity setups. Bi_2_Se_3_ was fabricated in our laboratory. TI is similar to graphene, possessing a large bandgap and a single Dirac cone, along with low saturation intensity, which serves as a potential pulse initiator. It behaves similarly to a metal at the outer layer but is insulating at the inner layer, with an energy bandgap of 0.2–0.3 eV. These unique properties suggest that TI exhibits excellent SA properties. In the past few years, works related to TI SA were published by various groups of researchers. Xu et al. [44] used a high modulation depth of 88% Bi_2_Se_3_ to generate mode-locked soliton with a pulse width of 579 fs at 1500 nm. Ahmad et al. [45] used Sb_2_Te_3_ SA to generate pulsing at the 2 um region. Another interesting finding was demonstrated using Sb_2_Te_3_ deposited on a microfiber to produce bound soliton with harmonic mode-locking [46]. A 22 nm wavelength tunable dissipative soliton was revealed by Wang et al. [47] using Bi_2_Te_3_ SA. Lately, a new member of TI, regarded as Bi_4_Br_4_ was reported by Liu et al. [48], with a high output power of 20 mW. They claimed that Bi_4_Br_4_ can withstand the high pump power. In this work, we observed a single soliton pulse with conventional Kelly sidebands from a simple cavity setup with Erbium fiber as the gain medium. The attainable repetition rate and pulse width was 23.3 MHz and 0.63 ps, respectively. When a section of single-mode fiber (SMF) was added to the existing cavity, we generated a bunch soliton. A total of 44 pulses were generated in the bunch soliton regime, and we observed interesting variations in the soliton sidebands. This peculiar soliton exhibited a repetition rate of 13 MHz and pulse width 1.52 ps. Our findings highlight the potential of Bi_2_Se_3_ as an effective SA in fiber lasers and contribute to the ongoing research regarding multiple soliton dynamics and soliton sideband variations.

## 2. Experimental Setup

**Figure 1:**
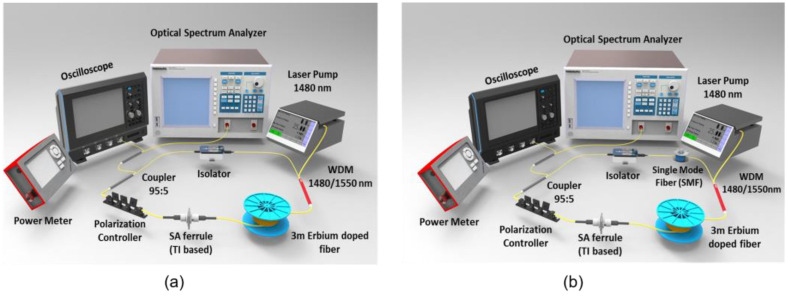
Experimental setup of the proposed soliton mode-locked EDFL with Bi_2_Se_3_ SA, (**a**) typical setup for single soliton pulse, (**b**) bunched soliton setup.

Figure 1a shows the configuration of the proposed TI-based mode-locked EDFL. The components used to construct the fiber laser were a wavelength division multiplexer (WDM), a polarization controller (PC), an isolator, and 95:5 coupler. The gain medium used was 3 m long EDF. The EDF had the following properties: core and cladding diameters of 4 μm and 125 μm, respectively, Erbium concentration of 2000 ppm and dispersion parameter of −21.64 ps/nm.km at wavelength 1550 nm. A 1480 nm laser diode was used to pump the EDF via 1480/1550 WDM. The remaining cavity consisted of a single mode fiber with a dispersion coefficient of 17 ps/nm.km at a wavelength of 1545 nm. The isolator forced the light to propagate in one single direction to enable lasing. PC was used to adjust the light polarization for optimum performance. A portion of light was extracted at the 5% port of 95:5 coupler for monitoring and laser characterization purposes. The Optical Spectrum Analyzer (OSA-Yokogawa AQ6370B) with a spectral resolution of 0.02 nm was employed to observe the pulse spectrum while the pulse repetition rate was recorded with an oscilloscope (Tektronix TDS3052C) in the form of an electrical signal via a 1.2 GHz bandwidth photodetector. Pulse width and pulse stability were measured using an auto-correlator and RF spectrum analyzer, respectively. The total cavity length was estimated to be 8.6 m long, while the total Group Delay Dispersion (GDD) was −0.19 ps^2^. The laser cavity was operating in the anomalous dispersion region.

We have previously published work on Bi_2_Se_3_ SA for Q-switching and mode-locking. In brief, 5 mg of Bi_2_Se_3_ was dissolved in 50 mL isopropyl alcohol by using a hotplate and a magnetic stirrer. After the mixing process, the solution was ultrasonicated to produce a stable composite solution. The optical deposition technique was adopted to transfer the solution to fiber ferrule. Its physical and chemical properties were characterized using FESEM, Raman Spectroscopy, and EDX. The size of the Bi_2_Se_3_ Raman peaks were approximately 3–4 nm. Our findings regarding its physical and chemical composition matched well with the work published by others. The dual optical power meter technique was used to characterize its saturable absorption. The nonlinear absorption behavior of SA can be modelled with the following equation:

(1) $$T\left(I\right)=1-∆Texp\left(-\frac{-I}{I_{sat}}\right)-\alpha_{ns}$$

where ∆T is the modulation depth, I is the input intensity, $I_{sat}$ is the saturation inten-sity, and $\alpha_{ns}$ is non-saturable absorption. It exhibited a modulation depth of 39.8% and a saturation intensity of 90.2 MW/cm^2^.

A detailed description of the preparation of Bi_2_Se_3_ SA can be viewed in reference [49]. The prepared Bi_2_Se_3_ SA was deposited on fiber ferrule and subsequently positioned in between EDF and PC. Figure 1b depicts another laser cavity configuration similar to Figure 1a, with the cavity length extended by using a section of 11.8 m long SMF. The overall cavity length was ~15.4 m, with a net anomalous total Group Delay Dispersion (GDD) of −0.39 ps^2^.

## 3. Results and Discussion

### 3.1. Single Soliton Pulse with Conventional Kelly Sidebands

**Figure 2:**
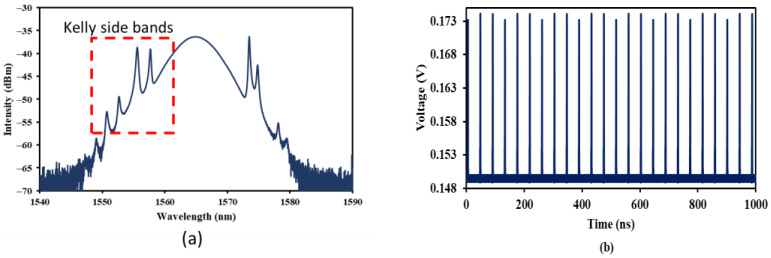

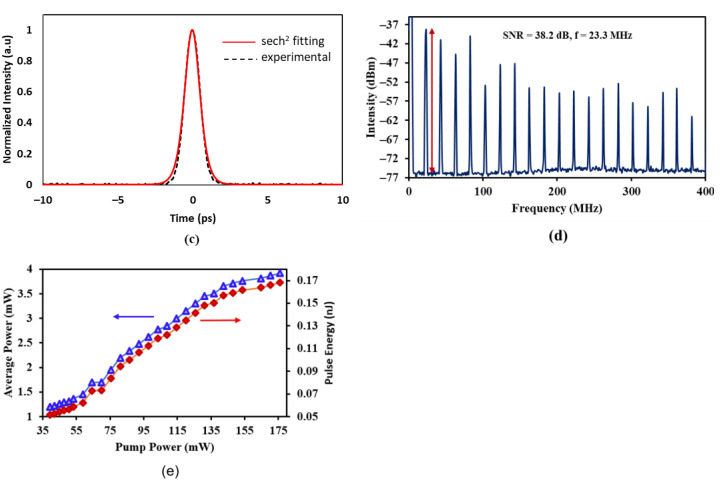
Characteristics of soliton pulse with Kelly sidebands at a pump power of 39.3 mW: (**a**) optical spectrum, (**b**) oscillation trace, (**c**) pulse width measurement, (**d**) RF spectrum, and (**e**) average output power and pulse energy against pump power.

With the high modulation depth of Bi_2_Se_3_ SA, self-starting mode locking was initiated at a relatively low pump power of 39.3 mW. The nonlinear saturable absorption characteristics of SA assisted the pulse formation and released short optical pulses. Figure 2a illustrates the soliton mode-locked spectrum captured at OSA at the pump power of 39.3 mW. The center wavelength was located at 1565 nm, with a peak power of −36.3 dBm. The 3 dB bandwidth was measured at 7.9 nm. The presence of spikes on the spectrum indicated that it was a soliton pulse. A total of four pairs of pronounced Kelly sidebands are visible on the spectrum. This is the typical pulse spectrum characteristics operating in the anomalous region. When the soliton was propagating in the fiber laser cavity, it produced dispersive waves due to periodic perturbations. The construction interference between soliton pulse and the dispersive waves formed the Kelly sidebands.

The temporal characteristics of the pulse are shown in Figure 2b,c. The oscillation trace of the soliton pulse in Figure 2b revealed that the peak-to-peak separation was 42.9 ns. The soliton pulse was repeating at the fundamental frequency of 23.3 MHz. The soliton pulse width (τ) was measured using an auto-correlator. Figure 2c describes the experimental autocorrelation (solid line) trace, which follows the sech^2^ fitting (dotted line) almost perfectly using the auto-correlator, with pulse width measurement τ taken at 0.63 ps. In Figure 2d, the stability of the pulse revealed an optical signal to noise ratio (OSNR) of more than 38 dB at a frequency of 23.3 MHz, according to the RF spectrum analyzer. The output power of the soliton mode-locked fiber laser measured with the power meter was recorded. Figure 2e summarizes the average power (refer blue arrow) and pulse energy (refer red arrow) with the increment of pump power from 39.3 mW to 175.8 mW. Both average power and pulse energy increased throughout the increment of pump power. The average power increased from 1.20 to 3.92 mW, while for pulse energy, it increased from 0.05 nJ to 0.17 nJ.

### 3.2. Bunched Soliton with Peak-Dip Sidebands

**Figure 3:**
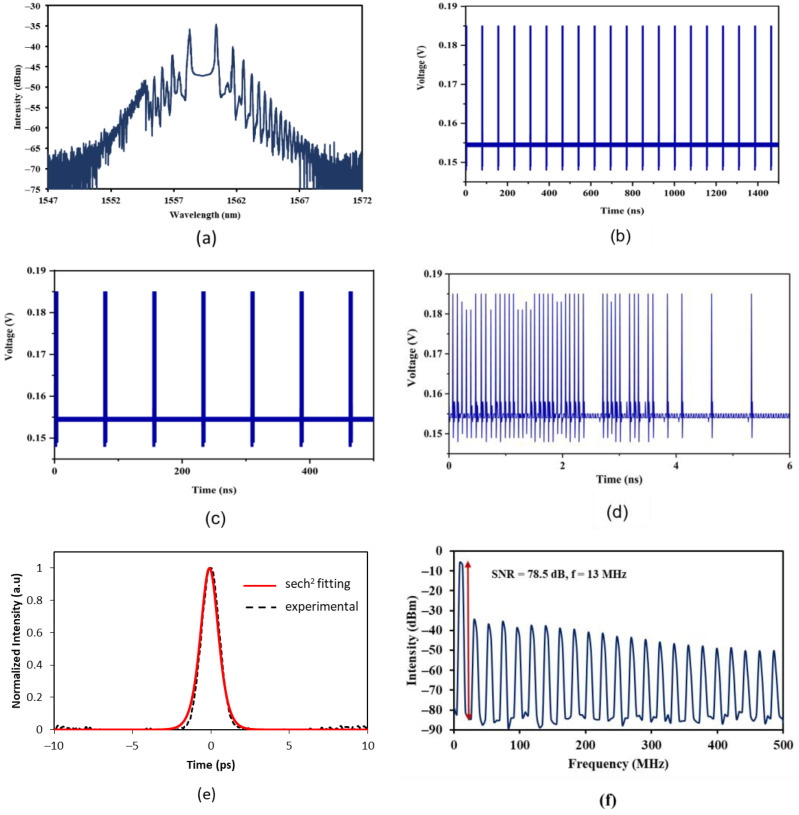

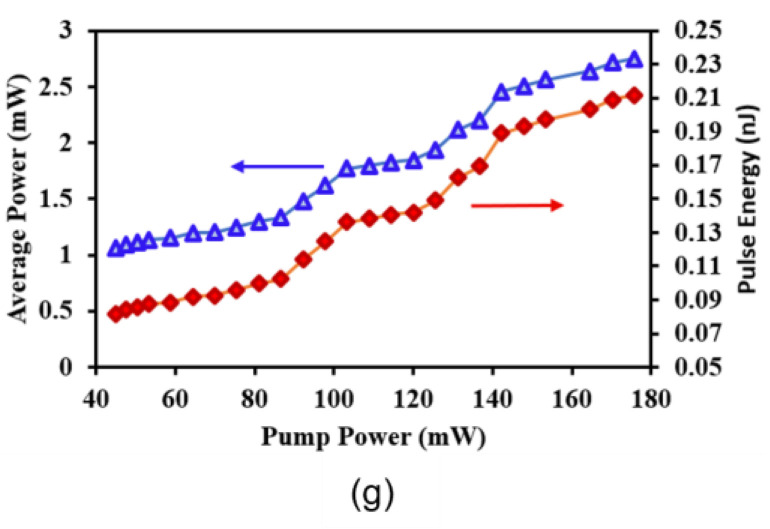
Output traces of soliton pulse with peak-dip sidebands: (**a**) OSA, (**b**) oscilloscope at 1500 ns time span, (**c**) oscilloscope at 500 ns time span, (**d**) oscilloscope at 6 ns time span, (**e**) autocorrelator, (**f**) RF spectrum analyzer, (**g**) average output power, and pulse energy against pump power; at the fixed pump power of 176 mW.

A section of SMF with a length of 11.8 m was added into the existing laser cavity configuration. A 1480 nm laser diode was gradually increased, the CW lasing changed into pulsed lasing when the pump power reached the threshold of 44.9 mW by carefully tuning the light polarization via PC. Compared to the previous set-up, the mode-locking threshold was slightly higher due to the insertion of SMF. The pump power was further increased to the maximum available pump power, and the mode-locked pulsing operation was maintained. Figure 3a shows the optical spectrum of the mode-locked pulse generated from the extended fiber laser cavity. Interestingly, the soliton mode-locked spectrum observed at OSA is different compared to the earlier setup. The spectrum was almost identical to the spectrum published by Han and Yun [17]. Peculiar sidebands were noticed and regarded as peak-dip sidebands. Many pairs of similar peak-dip sidebands could be located on the spectrum. Similar to Kelly sidebands, the peak sidebands were formed due to constructive interference between the soliton and dispersive waves. When the phase difference between the soliton and dispersive waves was 2πm (m is an integer), constructive interference took place, and this resulted in the formation of peak sidebands. On the other hand, when the phase difference was (2πm + 1), dip sidebands appeared due to destructive interference between the soliton pulse and dispersive wave. The center wavelength of the spectrum was located at 1559.4 nm and the 3-dB bandwidth of the spectrum is about 2.7 nm.

Figure 3b–d illustrates the oscillation trace of the mode-locked fiber laser at different time spans. According to the oscilloscope, the repetition rate was ascertained at 13 MHz, with peak-to-peak oscillation trace spacing being observed at 76.9 ns. It matched the cavity length with pulse oscillating at the fundamental frequency. An enhanced view of the soliton pulse can be seen in Figure 3c,d. The adjacent envelopes were evenly spaced apart. Figure 3d shows the zoom in view at a single oscillation trace at the time span of 6 ns. It was revealed that there were as many as 44 pulses bunched together in one single envelope of oscillation trace. The 44 pulses were not consistently spaced apart. In this extended laser cavity, the circulating soliton pulse accumulated additional nonlinear phase shift, and this caused the soliton pulse to break as the pulse obtained more energy once the mode-locking threshold was reached. The voltage intensity in Figure 3b–d has a non-zero pedestal. It is believed that the soliton pulse generated is a combination of bright and dark soliton pulse, at its vertical and horizontal axis. Dark solitons are characterized by their dip in intensity at the oscilloscope trace. The bunched soliton pulse width was measured using an autocorrelator, and Figure 3e displays the auto correlation trace (solid line) of 1.52 ps, if a sech^2^ pulse profile (dotted line) is assumed. Figure 3f shows the RF spectrum of the bunched soliton pulse. The OSNR was determined at 78 dB and at a frequency of 13 MHz. This signified that the fiber laser was stable in the laboratory environment. The average power and pulse energy were tabulated with respect to pump power as in Figure 3g. Both average power (refer blue arrow) and pulse energy (refer red arrow) increased with the pump power. The highest output power and pulse energy were measured at 2.75 mW and 0.211 nJ, respectively, at a pump power of 176 mW. This mode-locking process was weakly aided with a nonlinear polarization rotation effect (NPR) by using the additional SMF.

## 4. Conclusions

We synthesized Bi_2_Se_3_ SA in our laboratory and successfully proved that it was functioning perfectly as SA. Conventional soliton with Kelly sidebands and bunched soliton with peak-dip sidebands were obtained from two different laser cavity set ups. Both pulses were operating at the fundamental frequency—23.3 MHz and 13 MHz, respectively. There was a total of 44 pulses in a bunched soliton envelope—obtained from the cavity with extended SMF. The accumulated nonlinear phase shift caused the pulse to break into many soliton pulses in a single oscillation envelope. Constructive and destructive interference between soliton and dispersive waves occurred, and it resulted in peak-dip sidebands at the bunched soliton spectrum.

## Data Availability

Data are available for anyone who wants to look at it with reasonable arguments. Please contact the corresponding author.
